# Imidacloprid and chlorpyrifos insecticides impair migratory ability in a seed-eating songbird

**DOI:** 10.1038/s41598-017-15446-x

**Published:** 2017-11-09

**Authors:** Margaret L. Eng, Bridget J. M. Stutchbury, Christy A. Morrissey

**Affiliations:** 1 University of Saskatchewan, Toxicology Centre, Saskatoon, S7N 5B3 Canada; 2 York University, Department of Biology, Toronto, M3J 1P3 Canada; 3 University of Saskatchewan, Department of Biology, Saskatoon, S7N 5E2 Canada; 4 University of Saskatchewan, School of Environment and Sustainability, Saskatoon, S7N 5C8 Canada

## Abstract

Birds that travel long distances between their wintering and breeding grounds may be particularly susceptible to neurotoxic insecticides, but the influence of insecticides on migration ability is poorly understood. Following acute exposure to two widely used agricultural insecticides, imidacloprid (neonicotinoid) and chlorpyrifos (organophosphate), we compared effects on body mass, migratory activity and orientation in a seed-eating bird, the white-crowned sparrow (*Zonotrichia leucophrys*). During spring migration, sparrows were captured, held and dosed by gavage daily for 3 days with either the vehicle control, low (10% LD50) or high (25% LD50) doses of imidacloprid or chlorpyrifos and tested in migratory orientation trials pre-exposure, post-exposure and during recovery. Control birds maintained body mass and a seasonally appropriate northward orientation throughout the experiment. Imidacloprid dosed birds exhibited significant declines in fat stores and body mass (mean loss: −17% low, −25% high dose) and failed to orient correctly. Chlorpyrifos had no overt effects on mass but significantly impaired orientation. These results suggest that wild songbirds consuming the equivalent of just four imidacloprid-treated canola seeds or eight chlorpyrifos granules per day over 3 days could suffer impaired condition, migration delays and improper migratory direction, which could lead to increased risk of mortality or lost breeding opportunity.

## Introduction

Declines in migratory bird populations have been linked to a range of complex factors, including the large-scale application of agricultural pesticides^1,2^. Two of the most widely used classes of insecticides worldwide are the neonicotinoids, which entered the market in the 1990s^3^, and the older and more diverse chemistry of organophosphates, which increased in popularity following the regulation of organochlorine pesticides in the 1970s^4^. Both classes target cholinergic neurotransmission, although through different modes of action. Neonicotinoids are nicotinic acetylcholine receptor (nAChR) agonists^5^, and organophosphates are acetylcholinesterase (AChE) enzyme inhibitors^6^. Neonicotinoids typically bind more strongly to insect receptors than vertebrate receptors, and were thought to pose a lower risk for humans and non-target vertebrates than the organophosphates^3,5^. However, there is increasing evidence that both neonicotinoids and organophosphate insecticides can have direct and indirect effects on wildlife at environmentally relevant concentrations^7–13^_._

Birds that utilize agricultural landscapes may be exposed to insecticides through consumption of treated seeds, granules, or sprayed soils and prey items. Small migratory songbirds that regularly use farmland habitats as a stopover and refuelling source may be particularly susceptible to exposure and the negative effects of neurotoxic insecticides. Successful migration requires optimizing refueling and departure decisions, as well as accurate orientation^14,15^. Nocturnally migrating birds can use different compass systems (solar, stellar, magnetic) for orientation, and they can also use different environmental cues (e.g. olfactory, geomagnetic) for orientation and navigation^16^. Both the orientation and navigation systems in birds have a neural basis. The specific underlying neuronal mechanisms of long-distance migration are largely unknown^17^, but it is possible that neurotoxic insecticides that disrupt acetylcholine transmission could have effects on cognitive and motor functions that play important roles in refueling, orientation and navigation. Organophosphates and neonicotinoids have effects on survival, as well as sublethal neurophysiological and behavioural effects in birds, including impaired thermoregulation and food consumption^8–10,13,18,19^.

The energetic demands of long-distance flight and the negative fitness consequences of poor navigation and delays in arrival at the breeding grounds make migration one of the most vulnerable stages in a bird’s life cycle^20^. However, little is known about the direct impacts of pesticides on migration behaviour and success, which can be challenging to measure in the field. Migratory birds that use an area for rest and refueling are hard to track after they leave, and documentation of mortality events at the stopover site is difficult as affected birds are frequently removed by predators or scavengers within a short time frame^21–23^. Therefore, pesticide risks to migrating birds have likely been underestimated. There is growing evidence that pesticides and other toxicants disrupt flight efficiency and navigation in birds. Homing pigeons (*Columba livia*) exposed to carbamate (carbofuran, aldicarb) and organophosphate (chlorpyrifos) insecticides took significantly longer to return to their home loft after release^24,25^. There is also evidence that disruption of flight orientation is a sensitive endpoint of contaminant exposure in birds. A captive study on white-throated sparrows (*Zonotrichia albicollis*) during fall migration found that adult birds exposed to an organophosphate pesticide, acephate, were not able to establish a migratory direction, whereas control birds displayed a seasonally correct southward migratory direction^26^. More recently, a study on European starlings (*Sturnus vulgaris*) exposed to polychlorinated biphenyls (PCBs) during early development similarly showed delayed and incorrect orientation behaviour^27^. No study has yet tested if neonicotinoids disrupt bird migration.

Both imidacloprid and chlorpyrifos are currently widely used in North America. Imidacloprid is commonly applied as a seed treatment and chlorpyrifos is used both as a granular product and foliar spray on a wide variety of agricultural crops (e.g. corn, soy, fruit, oilseeds) as well as ornamental grasses and turf ^3,28^. In Canada, a proposal to phase-out imidacloprid is currently in the consultation period, and other neonicotinoids are under special review^29^. Imidacloprid regulations are also under review in Europe and the United States^30,31^. Registration of chlorpyrifos is currently under review in the United States and is scheduled for re-evaluation in Canada^32,33^. There is an urgent need for information on the potential effects of neurotoxic insecticides on seed-eating birds that forage in agricultural landscapes during migration. The objectives of this study were to assess effects of acute exposure to a representative neonicotinoid (imidacloprid) versus an organophosphate (chlorpyrifos) on migratory orientation, activity, and body mass in a model songbird species (the white-crowned sparrow, *Zonotrichia leucophrys*) caught at stopover sites during spring migration.

## Results

### Mass loss and mortality rates

**Figure 1:**
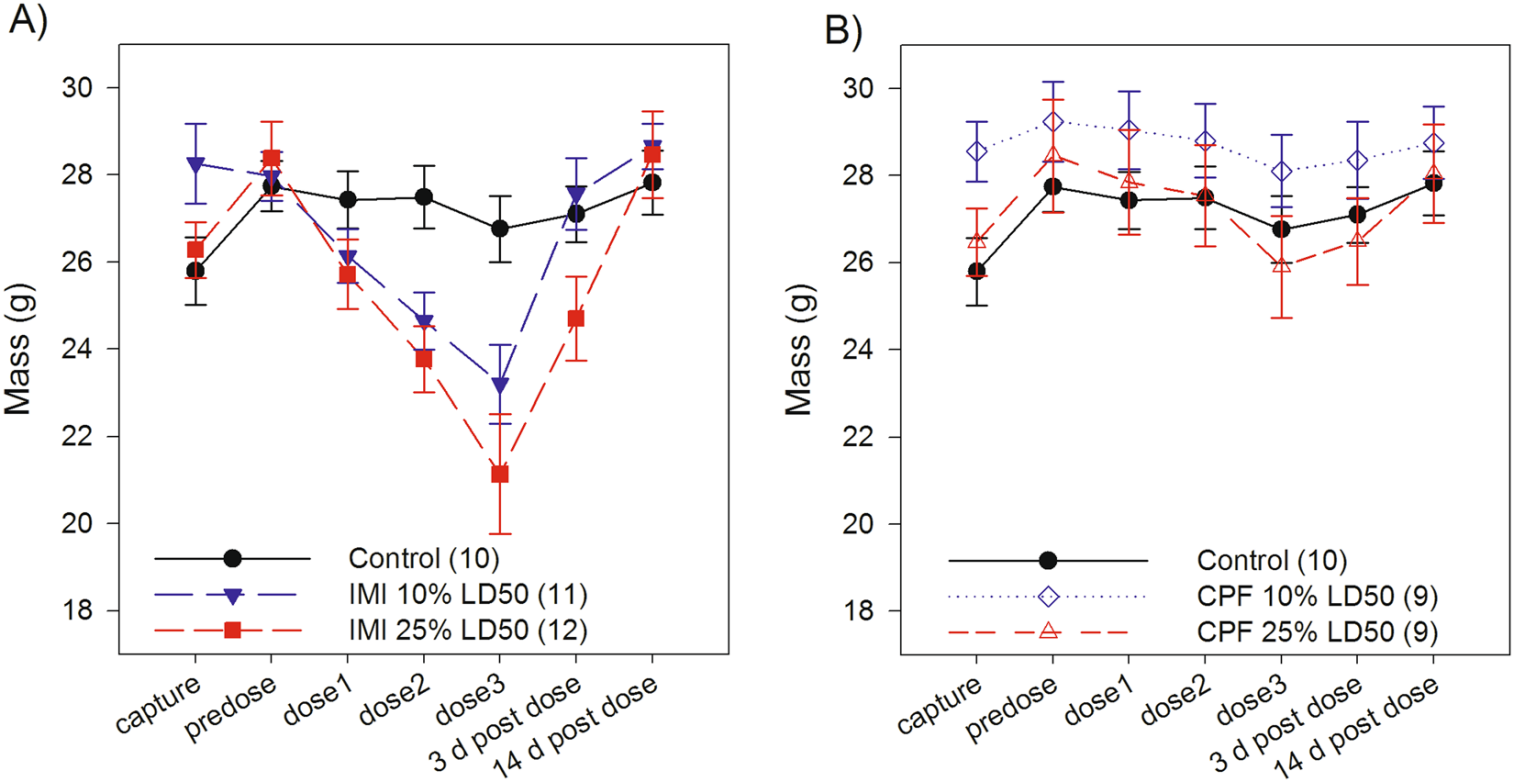
Change in average body mass of white-crowned sparrows exposed to imidacloprid (IMI), chlorpyrifos (CPF) or a vehicle control (sunflower oil), sample sizes in brackets. “Predose” represents body mass immediately before the first dose (see Fig. S1 for timeline). (**A**) Control birds maintained body mass for the duration of dosing (*p* > 0.213). IMI caused a significant reduction in body mass (dose*time *p* < 0.0001) in both the low (10% LD50) and high (25% LD50) IMI exposure groups starting after the first dose (predose vs. dose 1 mass: low dose *p* < 0.001, high dose *p* = 0.0001) and continued throughout the dosing period (predose vs. dose 3 mass: low dose *p* < 0.0001, high dose *p* < 0.0001). Body mass recovered in the low dose group within 3 days after the last dose (predose vs. 3 d post dose mass: low dose *p* = 0.156, high dose *p* < 0.0001) and in the high dose group within 2 weeks (*p* = 0.918). (**B**) Control birds are the same as those shown in (**A**). There was no effect of CPF on body mass. Body mass did change over time, with an increase during acclimation and a decrease during dosing; however, there was no significant interaction with dose (dose*time *p* = 0.187). Error bars represent standard error of the mean.

Birds in both the low and high imidacloprid exposure groups displayed acute signs of toxicity. While treatment was by daily oral gavage, birds reduced food consumption and experienced significant mass loss (Fig. 1). Overall, there was a significant change in body mass over time (F_6,157_ = 31.13, *p* < 0.0001), and there was a significant effect of dose on how body mass changed over time (dose*time interaction: F_12,157_ = 9.97, *p* < 0.0001). During the captive acclimation period, prior to any dosing, birds in the control and high dose group gained mass (*p* < 0.001) and birds in the low dose group maintained mass (*p* = 0.580). Control birds then maintained body mass for the duration of the experiment (*p* > 0.213). Body mass significantly declined compared to pre-dosing body mass within 24 hours of the first dose in both the low (*p* < 0.001) and high (*p* < 0.0001) imidacloprid dose groups, and continued to decline over the 3 days of dosing. After three days of exposure, the high dose imidacloprid group had lost on average 25.5% body mass and the low dose imidacloprid group had lost an average of 17% body mass, compared to 3.5% body mass loss in the control group. Body mass recovered in the low dose group (*p* = 0.156) within 3 days post-exposure, while the high dose group still had significantly reduced body mass (*p* < 0.0001) compared to the pre-dosing mass. Mass in both groups had recovered within 2 weeks following exposure (*p* ≥ 0.639). Overall females weighed less than males (F_1,29_ = 51.10, *p* < 0.0001); however, there was no interaction between dose and sex (F_2,27_ = 1.09, *p* = 0.352), indicating that males and females did not respond differently to exposure. Fat scores followed a similar pattern. Prior to dosing there was no difference in fat scores among treatment groups (χ^2^_2_ = 2.94, *p* = 0.230), and after 3 days of dosing body fat was significantly lower in dosed birds compared to control (χ^2^_2_ = 12.5, *p* = 0.002). These lower fat scores persisted to 3 days post-dosing (χ^2^_2_ = 8.61, *p* = 0.014), and then returned to control levels by 2 weeks post-dosing (χ^2^_2_ = 0.236, *p* = 0.889).

There was no statistical effect of imidacloprid treatment on mortality (Fisher’s exact test, *p* = 0.512). However, within 24 hours of receiving the third dose, 2 birds in the low imidacloprid dose group exhibited severe respiratory distress and were euthanized (18%), and 2 birds in the high imidacloprid dose group were found dead (17% mortality). None of the control birds died during the study. Symptomatic excess saliva in the crop and foaming at the mouth was observed in 2 low dose birds (18%) and 5 high dose birds (42%), compared to zero control birds (0%). The difference between treatment groups in the proportion of birds exhibiting these symptoms did not formally reach significance (Fisher’s exact test, p = 0.071). Although not quantified, treated birds also displayed general ataxia and lethargy during the dosing period even after a single oral dose. We did not weigh food to determine food consumption rates, however we observed that several high dose birds appeared to stop eating completely and low dose birds noticeably reduced food consumption during the dosing period.

In birds exposed to chlorpyrifos, there were no mortalities or overt signs of acute toxicity. There was a change in body mass over time (F_6,147_ = 8.30, *p* < 0.0001) with birds gaining weight following capture then losing weight during the 3 day dosing period, and then regaining weight during the recovery period, but there was no significant interaction between time and dose for body mass (F_12,147_ = 1.37, *p* = 0.187), indicating these changes in mass were similar for all dose groups. After 3 days of exposure, birds in the high chlorpyrifos exposure group lost an average of 9% of body mass, compared to 4% of body mass lost in the low chlorpyrifos group, and 3.5% of body mass lost in the controls. Average body mass across the whole experiment was not statistically different between the three groups (F_2,22_ = 0.52, *p* = 0.604). On average, females weighed less than males across all dose groups (F_1,22_ = 73.20, *p* < 0.0001). Fat scores were not different between chlorpyrifos treatment groups for any time point (*p* > 0.298). No mortality was observed in the chlorpyrifos treated birds.

### Migratory behaviour

**Figure 2:**
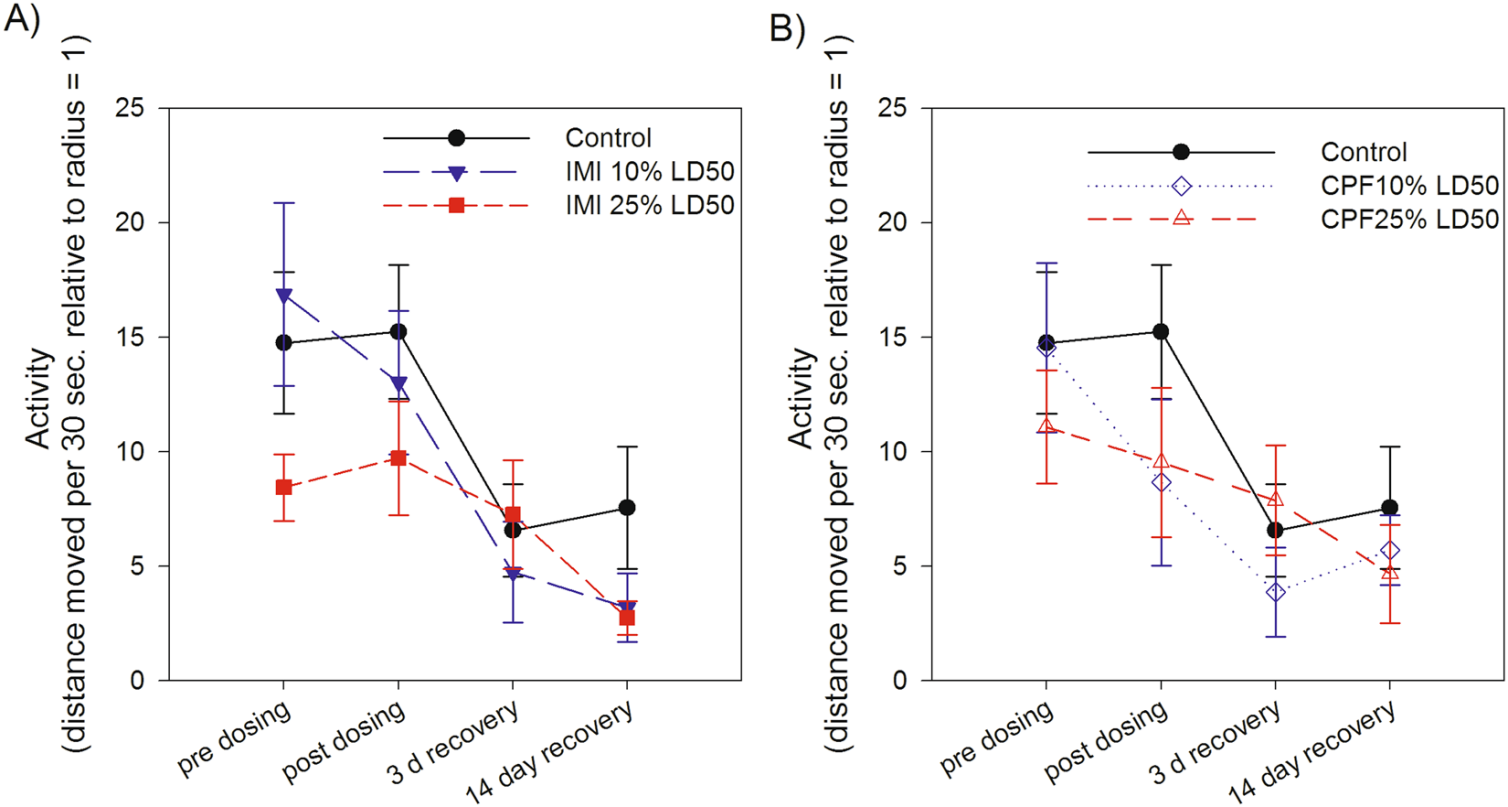
Migration activity, measured as the sum of distance moved over 30 second intervals relative to the central radius, of captive white-crowned sparrows during four Emlen funnel trials. Individual birds were tested pre and post dosing with (**A**) imidacloprid (IMI 10% or 25% LD50) or (**B**) chlorpyrifos (CPF 10% or 25% LD50) relative to controls. Activity decreased over time in all treatment groups (*p* < 0.0001), but there was no effect of imidacloprid or chlorpyrifos treatment on activity over time (dose*trial *p* = 0.183).

Migratory activity, measured as cumulative distance moved via outward hops in the funnels over 30 sec intervals, decreased over the course of experimental trials (i.e. time) (F_3,127_ = 20.45, *p* < 0.0001). There was no effect of imidacloprid or chlorpyrifos treatment (F_4,46_ = 0.73, *p* = 0.575) on activity level, and no interaction between treatment and trial (F_12,127_ = 1.38, *p* = 0.183) (Fig. 2). In addition, sex did not affect activity patterns (F_41_ = 0.06, *p* = 0.813) and the interaction between sex and treatment was also not significant (F_41_ = 0.44, *p* = 0.776).

**Figure 3:**
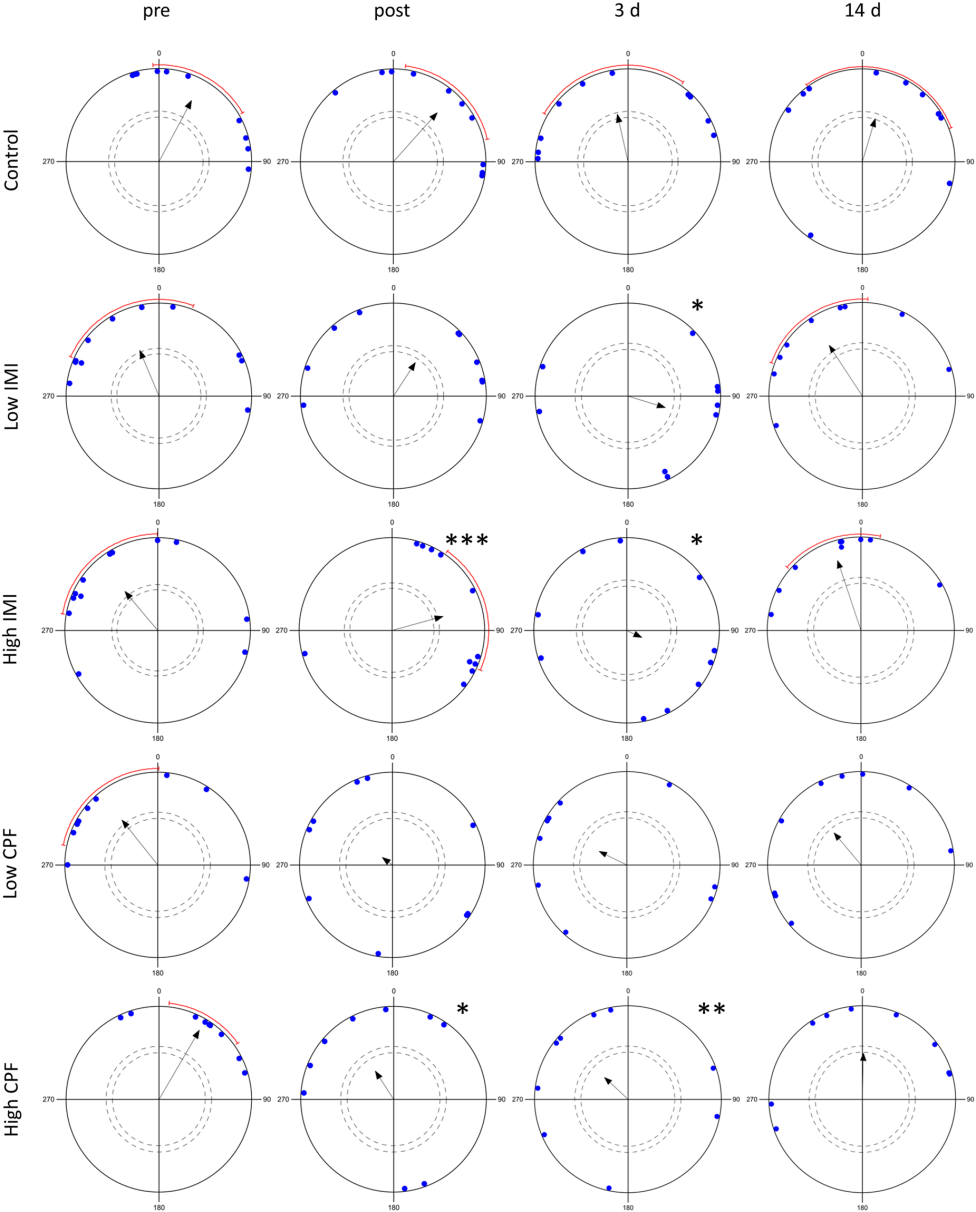
Migration orientation of white-crowned sparrows dosed with the vehicle control (sunflower oil), low (10% LD50) or high (25% LD50) concentrations of imidacloprid (IMI) or chlorpyrifos (CPF). Solid dots represent mean directions of individual birds. Arrows represent the mean orientation of all birds in a dose group for each time point, outer arc represents 95% confidence interval for each significant vector. Arrow length indicates how closely individuals are clustered around the mean (*r* = length of mean vector), and the dashed circles indicate the critical values for Rayleigh’s uniformity test at α = 0.05 (outer) and α = 0.10 (inner); vectors that pass these critical values are significant. Asterisks indicate difference in mean orientation direction compared to pre-dosing direction of birds within each treatment group for each time point following Hotelling’s paired test. Significance level: *** < 0.01, ** < 0.05, * < 0.1.

Pre-dosing, all treatment groups showed significant northward orientation (between 320° to 33°, *p* ≤ 0.038)) (Fig. 3). In the control group, the mean orientation direction did not change over repeated trials (Hotelling’s paired test *p* ≥ 0.22), although the strength of the orientation decreased with time (Rayleigh statistics; pre-dosing: Z = 5.57, *p* = 0.002; post-dosing: Z = 5.024, *p* = 0.004; 3 d recovery: Z = 2.765, *p* = 0.059; 14 d recovery: Z = 2.385, *p* = 0.09).

In the low dose imidacloprid group, birds failed to orient post-dosing (Z = 1.888, *p* = 0.152), but recovered by 2 weeks after exposure (Z = 3.812, *p* = 0.017). In the high imidacloprid dose group, the mean orientation direction changed post-dosing (75°) compared to pre-dosing (320°) (Hotelling’s F = 7.908, *p* = 0.01) and no orientation was observed 3 days after dosing ended (Z = 0.328, *p* = 0.73). Recovery of high dose imidacloprid birds to a northward orientation occurred at 2 weeks after exposure (Z = 5.628, *p* = 0.001).

Birds exposed to the low dose of chlorpyrifos showed no significant orientation post-dosing and no recovery of orientation at 3 and 14 days after dosing ended (*p ≥ *0.163). Similarly, birds exposed to the high dose of chlorpyrifos also showed no significant orientation post dosing (*p* > 0.108). High dose chlorpyrifos birds changed the mean direction of orientation at 3 days post-dosing (313°) compared to pre-dosing (30°) (F = 5.463, *p* = 0.037), although the 3 day post-dosing directional vector was not significant (*p* = 0.35). In contrast to the imidacloprid treatment groups, there was no recovery of migratory orientation at 2 weeks post-exposure for chlorpyrifos, whereas controls maintained seasonally appropriate northward orientation throughout.

## Discussion

White-crowned sparrows exposed to realistic concentrations of imidacloprid exhibited rapid and substantial declines in body mass and fat stores within 24 hours of exposure. Other symptoms of acute poisoning observed only in imidacloprid dosed birds include loss of appetite, excess saliva in the crop, and death. Both imidacloprid and chlorpyrifos disrupted orientation in captive trials during spring migration, while control birds maintained a seasonally appropriate northward bearing. Migration is a critical life stage, and birds that use agricultural habitats for refueling during migration may be particularly susceptible to exposure to neurotoxic insecticides. Species associated with grassland and agricultural landscapes are exhibiting severe population declines in North America^2,34,35^, and similar steep declines are occurring in European farmland species^36^. These declines have been linked to the intensification of agriculture and use of pesticides^1,37^. However, mechanistic captive and field studies examining pesticide effects to migratory birds are rare, and those that exist have largely focused on breeding birds. Here, we demonstrate how common and widely applied neurotoxic insecticides could affect the ability of birds to successfully migrate by reducing food consumption and body mass, and disrupting orientation.

The decrease in body mass and fat stores observed in imidacloprid exposed birds was most likely connected to decreased food consumption. Exposure was through oral gavage, and thus this lack of appetite is a direct effect of exposure rather than an aversion to treated seeds, which is in agreement with other dosing studies in birds that found reduced food consumption following imidacloprid exposure was due to post-ingestion distress rather than sensory repellency to coated seeds^38,39^. Prior to exposure, body mass was either stable or increasing, and body mass in the control group remained stable throughout the experiment, which excludes potential effects of captivity or seasonal changes on body mass. While mortality and the acute symptoms of poisoning were not statistically higher for imidacloprid exposed birds, we did not observe these symptoms in any other treatment group, and the lack of significance may be a factor of sample size. We also did not see any effects of chlorpyrifos at concentrations up to 7.4 μg/g (25% LD50) on body mass or any other symptoms of acute poisoning, while concentrations as low as 4.1 μg/g imidacloprid (10% of LD50) had clear effects. Despite neonicotinoids being a newer chemistry thought to have lower toxicity in non-target species, imidacloprid appeared to be more acutely toxic than chlorpyrifos in white-crowned sparrows. Other studies have also reported similar effects, including mass loss and mortality, in birds exposed to concentrations of imidacloprid that are likely to be encountered in the field^8,13^. Reduced food consumption and body mass at migratory stopover sites from even small doses of imidacloprid could have a negative impact on migrants whose ability to put on mass at stopover sites can constrain the overall speed and success of migration^14,40^, which consequently influences reproductive success and fitness^41,42^.

All dose groups showed decreased migratory activity over time. In the control group, the reduced activity and lower strength of orientation vectors over time could indicate a weakening of *zugunruhe* as the season progressed, as would be expected. The 14 day recovery Emlen trials took place in the first half of June, and in Gambel’s white-crowned sparrows the broad peak of migratory activity typically spans from March to June in the spring^43^. Both imidacloprid and chlorpyrifos had significant effects on the ability of birds to correctly orient, with the mean orientation direction significantly changed or being lost completely. These effects lasted ≥ 3 days from last exposure in all dosed birds. In imidacloprid exposed birds, there was evidence of recovery of orientation direction at 2 weeks post-exposure. In contrast, there was no recovery in the chlorpyrifos dose groups. If disruption of orientation ability were to occur at key time points during migration, navigation to the breeding grounds could be delayed or completely forgone. Changes in migratory orientation could affect the ability of birds to successfully reach the breeding or wintering grounds, or the length of time it takes to get there, and ultimately have long-term effects on reproduction and survival.

We observed differences in the magnitude and duration of the orientation disruption between the neonicotinoid and organophosphate insecticide groups that may be related to differences in the rate of detoxification, the strength of the nicotinic acetylcholine receptor binding, the reversibility of the AChE inhibition, in addition to other compound-specific mechanisms of action. Imidacloprid binds to the nicotinic acetylcholine receptor effectively holding the ion channel open and permitting continuous nerve cell stimulation^5^. Its activity and binding strength are believed to be lower in vertebrates than insects but songbirds appear susceptible likely due to receptor configuration enhancing binding affinity^5^. Chlorpyrifos (and its metabolite chlorpyrifos-oxon) irreversibly inhibits cholinesterase enzymes that are important for terminating nerve signal transmission by the neurotransmitter acetylcholine^6^. Both OPs and neonicotinoids can cause inhibitory effects on the autonomous nervous system with symptoms often related to increased lacrimation and salivation, neuromuscular weakness, impaired digestion and respiratory distress some of which were observed in the imidacloprid treated birds. The difference in orientation recovery between imidacloprid and chlorpyrifos treatments could possibly involve interactions between changes in food consumption and other compound-specific effects, as changes in food intake can affect locomotor activity and *zugunruhe* ^44,45^. In addition, detoxification of chlorpyrifos and imidacloprid can cause non-specific responses including oxidative stress^6,9,13^. Generation of reactive oxygen species carries high energetic costs and negative effects on aerobic performance, which could have implications for migration, a period of elevated energy demands.

Seed-eating birds that use agricultural landscapes have a real risk of exposure to insecticidal compounds at concentrations that could affect body condition and migratory behaviour. Doses used in the present study were selected to be sublethal (i.e. 10 and 25% of house sparrow LD50), and are at concentrations that could realistically be encountered by free-living birds occupying croplands. The low imidacloprid dose is equivalent to an average white-crowned sparrow (27 g) consuming ~4 treated canola seeds or less than a tenth of a corn seed, and the high dose is equivalent to consuming ~9 treated canola or 0.2 treated corn seeds, according to current US application rates (Table S2). Based on typical food consumption rates, this number of seeds would make up ≤ 1% of the daily sparrow diet (Table S2). Migrating birds frequently use agricultural fields sown with treated seeds^46^. Documented avoidance of imidacloprid treated seeds has been due to post-ingestion distress rather than sensory aversion to the seeds themselves^38,39^. This type of avoidance would not be protective of the serious effects that we have demonstrated can occur after ingesting just a few treated seeds.

A primary route of chlorpyrifos exposure is through consumption of granules mistaken as grit. Small seed-eating birds like sparrows have a high frequency of grit consumption, and their preferred grit size closely overlaps with the particle size used for granular pesticides^47^. The low and high chlorpyrifos doses would be equivalent to consuming ~8 or 21 granules, respectively (Table S2), which is within the range of the number of pesticide granules expected to be mistakenly consumed as grit. In a study assessing grit preference, house sparrows given *ad libitum* access to a choice of grit (silica or heat-treated clay), consumed an estimated 312 grit particles per day, of which 21 (7%) were made out of the same heat-treated clay used for chlorpyrifos granules^47,49^. When feeding in agricultural fields, the accessibility of chlorpyrifos granules may lead to sufficiently high ingestion rates to cause impaired migration.

Agricultural landscapes make up a significant portion of the land cover and bird habitat globally^35,49^, and more work is needed to evaluate the influence of insecticide use on migratory bird populations. This is the first study to compare the direct toxicity of neonicotinoids with older organophosphate chemistries. Uniquely, we were able to assess sublethal effects in wild songbirds during a migration event. We found acute effects of imidacloprid but not chlorpyrifos on body mass and fat stores, and negative consequences of both imidacloprid and chlorpyrifos in captive orientation trials. Even where recovery is possible, observed sublethal effects on fueling and migration behaviour could contribute to delays or failures of birds reaching their breeding grounds and may have significant fitness implications. These results highlight the importance of considering endpoints other than mortality when assessing the potential impact of pesticides on wildlife. Future studies are still needed to corroborate effects of neurotoxic insecticides on migration in free-living birds at an ecologically relevant scale, potentially using advanced tracking technologies now widely available.

## Methods

### Study species, capture and captive housing

White-crowned sparrows can migrate thousands of kilometers between their wintering and breeding grounds, and are frequently used as models in migration research (e.g.^45,50^). They are granivores, and have the potential to be directly exposed to insecticides through consumption of treated seeds or granules. These characteristics make them a valuable test species for providing insight into the potential effects of neurotoxic insecticides on migratory birds occupying agricultural fields during migration. In May 2016, A total of 57 Gambel’s white-crowned sparrows (*Z*. *l*. *gambelii)* were captured in mist nets or sparrow traps near Saskatoon, Saskatchewan (SK) (51° 58’ 27” N 106° 42’ 6” W) using playbacks or baited seed feeders as attractants. Birds were transported to the Facility for Applied Avian Research at the University of Saskatchewan, where they were held in cages (24”L × 24”W × 24”H) in an outdoor pen, with 2 to 3 birds per cage. Birds were provided with water and a mixture of millet, black oil sunflower seeds, and poultry starter crumbles (Proform 26%) *ad libitum*. Prior to dosing, birds were held for ~2 weeks to allow for acclimation to captivity and to increase the likelihood that they were in a migratory state, which was assessed through video monitoring of nighttime activity, increasing fat scores, and weight gain. At the end of the experiment, all birds were humanely euthanized by CO_2_ asphyxiation, gonadal sex was determined, and tissues were collected for future analysis. Research protocols were in compliance with the Canadian Council on Animal Care guidelines and approved by the University of Saskatchewan Animal Care Committee (AUP 20110043), and conducted under Canadian Wildlife Service Scientific Permit 15SKSC005. The timeline for experimental procedures (Emlen funnel trials, dosing, and body mass monitoring) is illustrated in Figure S1.

### Emlen funnel trials

Migratory behaviour was monitored with nocturnal Emlen funnel trials^51^, using flower pots as modified Emlen funnels as previously described^27^ (Fig. S2). Emlen funnels are circular in shape with sloped sides that force the bird to return to the center after an activity bout, while being wide enough so that outward movement can be detected by overhead cameras. Trials were conducted in an open field without any visual cues on evenings with clear skies.

Funnels were placed upright with birds having a clear view of the sky at least 35 minutes prior to sunset, and were left undisturbed for at least 5 minutes before recording data. Video of funnel trials was recorded from approximately 30 minutes before to 30 minutes after sunset using digital video cameras (ADS-180, Swann Communications) suspended 10 ft above the funnels. Each camera recorded movements from six funnels simultaneously onto a digital video recorder (DVR8-2550, Swann Communications). Each bird was tested in four trials over the course of the experiment: 1) pre-dosing: after the acclimation period to confirm birds are in a state of *zugunruhe* (migratory restlessness), 2) post-dosing: ~10 hours after the final dose, 3) 3 d recovery: 3 days after the post-dosing Emlen trial to test acute recovery, 4) 14 d recovery: 14 days after the post-dosing Emlen trial to test long-term recovery.

### Dosing

51 sparrows that were confirmed to be in a state of *zugunruhe* were randomly assigned to one of four dose groups or the control groups. Dosing started the morning after the first (pre-dosing) Emlen trial. Birds were orally dosed between 09:00 and 11:00 by gavage with 4.1 µg imidacloprid/g bw/day (IMI low; n = 11; 7 male, 4 female), 10.25 µg imidacloprid/g bw/day (IMI high; n = 12, 6 male, 6 female), 2.9 µg chlorpyrifos/g bw/day (CPF low; n = 9; 6 male, 3 female), 7.4 µg chlorpyrifos/g bw/day (CPF high; n = 9; 4 male, 5 female) (nominal concentrations), or a vehicle control (food-grade organic sunflower oil, Compliments brand, Sobeys Canada; n = 10; 4 male, 6 female) once per day for 3 days (acute 72 hour exposure) at a volume of 10 µl dosing solution/g bw. Dosing concentrations were selected through pilot studies conducted the previous year, and based on published values for the median lethal dose (LD50) for house sparrows (*Passer domesticus*) for each compound^28,52,53^. Therefore, these doses correspond to 10% or 25% of the predicted house sparrow LD50. Dosing solutions were made by dissolving technical grade imidacloprid (Sigma Aldrich 37894) or chlorpyrifos (Dursban, Sigma Aldrich 442573) in a small volume of acetone, then diluting with sunflower oil. Solutions were stirred overnight to evaporate off acetone, and stored in amber glass bottles in the dark for the duration of the study.

### Monitoring

Body mass (g) and fat scores (0 to 5) were measured between 09:00 and 11:00 at capture, before each dose, the morning after the last dose, the morning after the 3 day recovery Emlen trial, and the morning after the 14 day recovery Emlen trial. At capture and at the completion of the experiment, structural measures (tarsus, wing length, tail length, bill length, head-bill length) were taken.

### Chemical Analysis

Concentrations of imidacloprid and chlorpyrifos in dosing solutions were confirmed by liquid chromatography tandem mass spectrometry (LC/MS/MS) analyses at the National Hydrology Research Centre, Environment and Climate Change Canada, Saskatoon, SK.

Dosing solution samples (500 µL) were transferred to a 50 ml centrifuge tube containing 10 ml Milli-Q water. An additional 4.5 ml Milli-Q water was added along with 15 ml of acetonitrile containing 1% acetic acid, then briefly vortexed. AOAC QuEChERS extraction salts (Agilent-#5982-6755) were added and the tube shaken vigorously for 2 minutes. After mixing, the solution was centrifuged (5 min @ 5000 rpm). An EMR Lipid dSPE tube (Agilent-#5982-1010) was prepared by adding 5 ml Milli-Q water and briefly vortexing. 5 mL of the QuEChERS extract (acetonitrile layer) from above was transferred to the EMR lipid dSPE tube, vortexed thoroughly, shaken for 2 minutes followed by 5 minutes of centrifuging at 5000 rpm. 5 mL of this supernatant was transferred to an EMR-Lipid polish tube (Agilent-#5982-0101) and immediately vortexed then centrifuged for 5 min @5000 rpm. 200 µL of the acetonitrile layer was transferred into a 1.8 ml amber glass LC vial containing 800 µL of Milli-Q water followed by subsequent dilutions to bring the concentration within the calibration curve. 20uL of a 2.5 mg/L solution of internal standard (d4-imidacloprid, CDN Isotopes, Pointe-Claire, Quebec, CA) was added to the LC vial and vortex mixed immediately prior to instrumental analysis.

For the LC/MS/MS analysis, a Waters 2695 Alliance HPLC system (Waters Corp., Milford, MA), consisting of a solvent degassing unit, pump and autosampler, was used with a Waters XTerra MS-C18 (3.5 µm dia. particle size) column (2.1–100 mm) (Waters Corp., Milford, MA) at 30 °C. Isocratic elution of the analytes was achieved with an 25:75 (v/v) mix of solvent A (100% water and 0.1% formic acid) and solvent B (100% acetonitrile and 0.1% formic acid) respectively at a flow rate of 200 µL/minute. The run time was 10 min and the sample injection volume was 20 µL. Chlorpyrifos and imidacloprid were quantified (internal standard method) and their concentrations confirmed using the Micromass Quattro Ultima triple quadrupole mass spectrometer (Waters Corp., Milford, MA) equipped with an electrospray ionization interface set to positive ion mode. Ionization and MS/ MS conditions were optimized by infusing a 0.5 mg/L solution of each insecticide into the ion source in a 50:50 (v/v) acetonitrile:water solution containing 0.1% formic acid. MRM transitions, selected from the product ion scan and optimal cone voltages and collision energies for each analyte are provided in Table S1. Other instrumental settings were as follows: source temperature, 90 °C; capillary voltage, 3.95 kV; desolvation temperature, 220 °C; nitrogen desolvation gas flow rate, 487 L/h; nitrogen cone gas flow rate, 153 L/h; nitrogen nebulizer gas flow rate was at maximum flow; and multiplier voltage, 700 V. Argon was used as the collision gas at a pressure which increased the Pirani gauge reading to 2.10 × 10E-4 torr. Resolution was set to achieve unit mass resolution for quadrupole 1 and approximately 2 amu resolution for quadrupole 3.

Analytical standards of imidacloprid and chlorpyrifos were purchased from Chem Service (West Chester,PA, USA). A five-level calibration curve (5 to 100 µg/L) was analyzed before and after each batch of samples which also contained a laboratory or field blank and a fortified sample. Intermediate (1.0 mg/L) and working calibration standards were prepared fresh daily in Milli-Q water by serially diluting a substock containing each analyte at 100 mg/L in pure acetonitrile. This was necessary as it was observed that chlorpyrifos standards made in water were subject to degradation but were found stable in acetonitrile. All dosing solutions, blanks, and QC samples were run in triplicate. Measured concentrations of imidacloprid were 1.07 µg/µL ± 2%RSD and 0.40 µg/µL ± 11.8%RSD (nominal concentrations 1.03 µg/µL and 0.41 µg/µL). Measured concentrations of chlorpyrifos were 0.65 µg/µL ± 12.7%RSD and 0.23 µg/µL ± 5.7%RSD (nominal 0.74 and 0.29 µg/µL). Mean recoveries from sunflower oil fortified with chlorpyrifos at 0.51 µg/µL (n = 3) was 62.5% ± 3.1%RSD while sunflower oil fortified with imidacloprid at 0.72 µg/µL (n = 3) was 80.9% ± 2.7%RSD. Vehicle control oil and all blanks had no detectable levels of chlorpyrifos or imidacloprid.

### Statistical analysis

Statistical analysis for effects on body mass and activity was completed using SAS 9.4. Activity was square root-transformed to meet assumptions of normality. Comparisons between dose groups over time were made using linear mixed models (proc MIXED) with bird ID as a repeated subject effect, and fixed effects of dose, time and dose*time. Sex and the sex*treatment interaction were included as fixed factors, but non-significant terms were removed from the final model. Tests for differences between means were adjusted for multiple comparisons using the Tukey-Kramer method. Fat scores were compared among dose groups using the Kruskal-Wallis test. Fisher’s Exact Test was used to assess effects of dose on survival. Significance level was set at α = 0.05

BirdOriTrack software^54^ was used to analyze videos and determine activity (distance each bird moved every 30 seconds during valid hops, relative to the radius of the funnel [radius = 1]) and mean orientation for each bird in each trial. A hop was considered valid when the movement was ≥ 0.5 of the radius from the centre of the funnel. Videos were reviewed immediately following the pre-dosing Emlen funnel trial to qualitatively determine bird activity, as only active birds that appeared to be in a state of *zugunruhe* continued on in the experiment for dosing and further trials. Circular statistics were analyzed using Oriana 4.02 (Kovach Computing Services). The strength of orientation was assessed using Rayleigh’s uniformity test (Rayleigh Z statistic). Significant directional (vs. uniform) orientation was tested within each treatment group at each time point. The difference in the mean orientation direction of birds between pre-dosing and later time points within each dose group was compared using Hotelling’s paired test.

### Data Availability

The datasets generated during and/or analysed during the current study are available from the corresponding author on reasonable request.

## Electronic supplementary material

Supplementary Information
