# Supplementary material for: Imidacloprid and chlorpyrifos insecticides impair migratory ability in a seed-eating songbird
Source: Sci Rep. 2017 Nov 9;7:15176. doi: 10.1038/s41598-017-15446-x (PMC5680183; doi:10.1038/s41598-017-15446-x)
Supplement: Supplementary file 1 — Supplementary Information [file 41598_2017_15446_MOESM1_ESM.pdf]

Supplementary Materials

Imidacloprid and chlorpyrifos insecticides impair migratory ability in a seed-eating songbird

Margaret L. Eng[1], Bridget J.M. Stutchbury[2], and Christy A. Morrissey[3,4,*]

[1]University of Saskatchewan, Toxicology Centre, Saskatoon, S7N 5B3, Canada

[2]York University, Department of Biology, Toronto, M3J 1P3, Canada

[3]University of Saskatchewan, Department of Biology, Saskatoon, S7N 5E2, Canada

[4]University of Saskatchewan, School of Environment and Sustainability, Saskatoon, S7N 5C8, Canada

*christy.morrissey@usask.ca

Table S1: MRM transitions, selected from the product ion scan and optimal cone voltages and collision energies for imidacloprid and chlorpyrifos

Analyte	MRM (m/z)	Cone voltage (V)	Collision Energy (eV)	Retention time (min)
Imidacloprid	255.80>174.90<br>255.80>209.00	27.00<br>27.00	18.00<br>18.00	1.51
Imidacloprid-d_4	259.80>178.90<br>259.80>213.00	27.00<br>27.00	18.00<br>18.00	1.49
Chlorpyrifos	349.80>152.90<br>349.80>197.80	6.00<br>6.00	26.00<br>26.00	4.75

Table S2. Comparison of Imidacloprid low (4.1 μg IMI/g bw/day) and high (10.25 μg IMI/g bw/day) doses to potential exposure through seed consumption based on current application rates in Canada[a] and the US[b], and calculation of the number of Lorsban 15G granules to make up chlorpyrifos low (2.9 μg CPF/g bw/day) and high (7.4 μg CPF/g bw/day) doses. Estimates are based on an average white-crowned sparrow body mass of 27 g, with average seed consumption 7.9 g/day[c].

	Cereals[e] Canada	Cereals US	Canola Canada	Canola US	Corn Canada	Corn US	Sunflower US	CPF granules[f]
average mass of individual seed (g)[d]	0.035	0.035	0.003	0.003	0.38	0.38	0.05	.000064
application rate	up to 30 g a.i./100kg seed	up to 2.4 floz/100lbs	802 g a.i./100kg seed	up to 25.6 fl oz/100lbs	48 g a.i./80,000 seeds	up to 1.34 mg/kernal	up to 0.5 mg a.i./seed	n/a
mg active ingredient (a.i.)/g seed	0.3	0.93	8.02	9.97	1.58	3.53	10	150
mg a.i. per individual seed	0.0105	0.033	0.024	0.03	0.6	1.34	0.5	0.0096
number of seeds consumed per day (if 100% of diet)	225.7	225.7	2633.33	2633.3	20.79	20.8	158	n/a
mg IMI per day if diet 100% treated seeds	2.37	7.4	63.2	78.8	12.47	27.9	79	n/a
number of seeds to consume equivalent to high dose	26.4	8.5	11.54	9.3	0.46	0.21	0.6	8.2
% of diet made up of treated seeds to consume equivalent to high dose	11.7	3.8	0.44	0.4	2.21	1.01	0.4	n/a
number of seeds to consume equivalent to low dose	10.6	3.4	4.625	3.7	0.19	0.08	0.2	20.8
% of diet made up of treated seeds to consume equivalent to low dose	4.7	1.5	0.18	0.1	0.91	0.4	0.1	n/a

[a]Health Canada PVRD2016-20 appendix IIa[1]
[b]Gaucho 600 flowable, US EPA reg 264-968
[c]food consumption values from[2]
[d]Seed masses from[3]
[e]wheat, barley, oats, rye, triticale
[f]granule mass from[4]

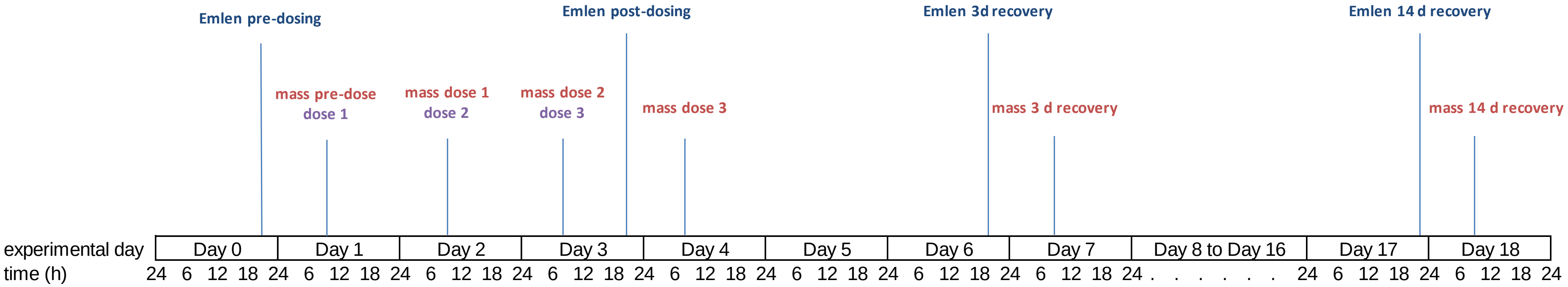

Fig. S1. Timeline of experimental procedures, starting after birds had acclimatized to captivity and were confirmed to be in a state of *zugunruhe* (Day 0). Emlen funnel trials were recorded for approximately 30 minutes before to 30 minutes after sunset. Birds were weighed immediately before dosing, and the morning after each Emlen funnel trial. All weighing and dosing took place between 09:00 and 11:00.

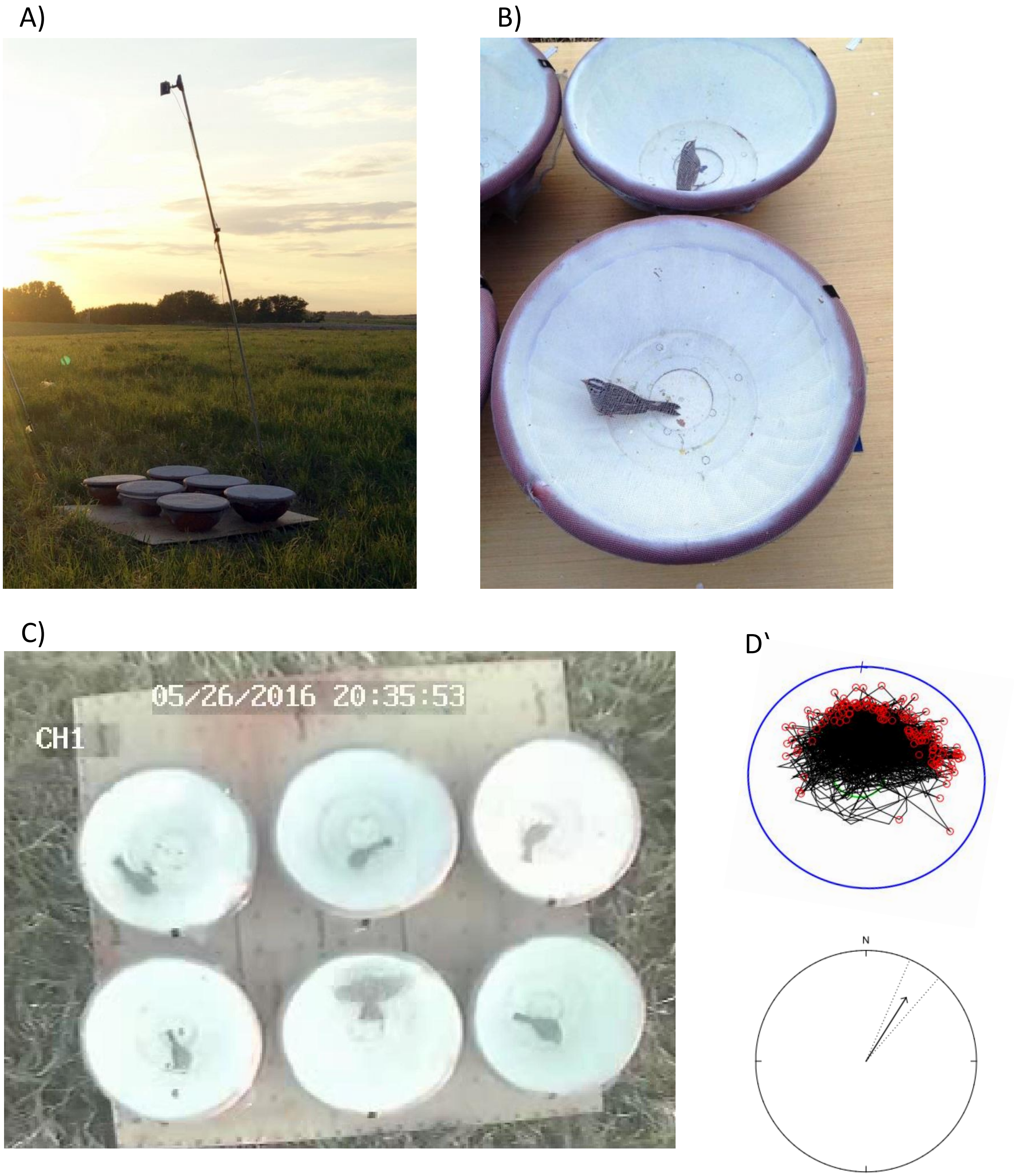

Fig. S2. Emlen funnel trial setup. A) Camera is suspended on 3 m pole above Emlen funnels. B) Funnels are made out of plastic flower pots with sloping sides. A mesh screen covers the top, and birds are placed in the funnel through a covered hole in the centre of the base. When a bird hops in the direction it wants to fly, it is forced to return to centre. C) Video from one camera, which can record 6 funnels at once. D) Output from BirdOriTrack tracks valid hops (movement ≥ 0.5 of the radius from centre of funnel) and calculates mean orientation for each bird in each trial.

References

1 Health Canada. Imidacloprid: Proposed Re-evaluation Decision. *Pest Management Regulatory Agency* **PRVD2016-20** (2016).

2 Ramenofsky, M., Agatsuma, R. & Ramfar, T. Environmental conditions affect the behavior of captive, migratory white-crowned sparrows. *The Condor* **110**, 658-671 (2008).

3 Mineau, P. & Palmer, C. The impact of the nation's most widely used insecticides on birds. *American Bird Conservancy* (2013).

4 Solomon, K. *et al.* Chlorpyrifos: ecotoxicological risk assessment for birds and mammals in corn agroecosystems. *Human and Ecological Risk Assessment* **7**, 497-632 (2001).
